# Comparative genomic analyses highlight the contribution of pseudogenized protein-coding genes to human lincRNAs

**DOI:** 10.1186/s12864-017-4156-x

**Published:** 2017-10-16

**Authors:** Wan-Hsin Liu, Zing Tsung-Yeh Tsai, Huai-Kuang Tsai

**Affiliations:** 1 Institute of Information Science, Academia Sinica, Taipei, 115 Taiwan; 2 Bioinformatics Program, Taiwan International Graduate Program, Academia Sinica, Taipei, 115 Taiwan; 3 Institute of Bioinformatics and Systems Biology, National Chiao Tung University, Hsinchu, 300 Taiwan; 4 Department of Plant Biology, Michigan State University, East Lansing, MI 48824 USA

**Keywords:** Long intergenic noncoding RNAs, Pseudogenization, Transposable element, Competing endogenous RNA, Syntenic analysis

## Abstract

**Background:**

The regulatory roles of long intergenic noncoding RNAs (lincRNAs) in humans have been revealed through the use of advanced sequencing technology. Recently, three possible scenarios of lincRNA origins have been proposed: de novo origination from intergenic regions, duplication from other long noncoding RNAs, and pseudogenization from protein-coding genes. The first two scenarios are largely studied and supported, yet few studies focused on the evolution from pseudogenized protein-coding sequence to lincRNA. Due to the non-mutually exclusive nature of these three scenarios and the need of systematic investigation of lincRNA origination, we conducted a comparative genomics study to investigate the evolution of human lincRNAs.

**Results:**

Combining with syntenic analysis and stringent Blastn *e*-value cutoff, we found that the majority of lincRNAs are aligned to intergenic regions of other species. Interestingly, 193 human lincRNAs could have protein-coding orthologs in at least two of nine vertebrates. Transposable elements in these conserved regions in human genome are much less than expectation. Moreover, 19% of these lincRNAs have overlaps with or are close to pseudogenes in the human genome.

**Conclusions:**

We suggest that a notable portion of lincRNAs could be derived from pseudogenized protein-coding genes. Furthermore, based on our computational analysis, we hypothesize that a subset of these lincRNAs could have potential to regulate their paralogs by functioning as competing endogenous RNAs. Our results provide evolutionary evidence of the relationship between human lincRNAs and protein-coding genes.

**Electronic supplementary material:**

The online version of this article (10.1186/s12864-017-4156-x) contains supplementary material, which is available to authorized users.

## Background

Long intergenic non-coding RNAs (lincRNAs) are a subclass of non-coding RNAs, which are longer than 200 nucleotides and locate between protein-coding genes. The advance of sequencing technology has recently revealed that lincRNAs are present in various aspects of the transcriptome and largely transcribed in many species from invertebrates to humans [1–3]. LincRNAs participate the regulation of many biological processes, such as the development of neuron components [3], gene expression [4, 5], and carcinogenesis [6]. Although the functions of lincRNAs are gradually explored, the mechanism generating lincRNA has not attained a conclusion. Considering the origination of lincRNAs would increase the regulatory complexity and impact several biological processes, it is intriguing to understand the evolution of lincRNAs and their evolutionary mechanisms.

Currently, three non-mutually exclusive possible mechanisms of lincRNAs origination have been proposed [1, 2, 7]. First, lincRNAs could have evolved from the duplication of other long non-coding RNAs (lncRNAs). Second, lincRNAs could have evolved de novo, where their sequences could be previously noncoding or derived from transposable elements (TEs). Third, lincRNAs could have evolved from pseudogenization of protein-coding gene sequences. A well-studied lincRNA, *Xist*, which plays an important role in X chromosome inactivation [8, 9], is regarded as having originated from a protein-coding gene, *Lnx3*, as shown to contain the debris of the sequence of *Lnx3* [10].

TE has been suggested as one of the major driving forces of lincRNA evolution. The capability of TE to transfer sequence to different regions across the genome allows new transcripts by providing valid regulatory sequences. Regulatory sequences such as promoter, transcription start site, enhancer, and splicing site can lead to transcription of a novel RNA [11] or process a precursor RNA into a stable transcript [12, 13]. A recent study has estimated about 10% of the human lncRNA transcripts originated from long terminal repeats [11]. Moreover, many mature lncRNAs have been identified as entirely composed of endogenous retroviral sequences [11]. In addition to the hypotheses that regulatory sequences may derive lncRNAs, studies also found a substantial fraction of lncRNAs contains TE-derived sequences [14, 15], indicating a close association between TE and lncRNA.

However, due to the low conservation of lincRNAs sequences comparing to the mRNAs sequences, the number of studies about the relationship between lincRNAs and protein pseudogenization is more limited. In addition, the high TE composition in the exonic regions of lincRNAs (i.e. TEs inserted in the pseudogenes before and/or after the birth of the lincRNA) could also lead to the underestimation of the contribution of protein-coding gene pseudogenization in lincRNA origination [11]. Therefore, a detailed investigation of lincRNAs originated from protein-coding gene pseudogenization is needed. Two key questions of the extents of the pseudogenized protein-coding genes contribute to lincRNA origination and whether there are resulting regulations of these lincRNAs should be addressed.

Pseudogenes have two different types, i.e. duplicated pseudogene and unitary pseudogene, where each type originate by different mechanisms and have distinct characteristics [16]. A duplicated pseudogene is a copy of a gene that has been modified during and/or after duplication leading to loss of gene function. Alternatively, unitary pseudogene is a functional gene becoming disabled instead of derived and disabled from a duplicated copy of a gene. Thus, the lincRNAs originated from protein-coding gene pseudogenization might follow these two different evolutionary trajectories, which has not been investigated thoroughly thus far.

In this study, we focused on the human lincRNAs that might derive from protein-coding gene sequences. The potential origination for human lincRNAs were investigated by identifying its homologous sequences across nine vertebrate species. We analyzed sequence compositions and genomic locations of these putative orthologs to understand the origination of human lincRNAs and the biogenesis components that contributed to their origination. Our results show most of the human lincRNAs have putative orthologs in at least two other vertebrates. Interestingly, although the majority are located in intergenic regions as expected, certain portions of these putative orthologs are partially or even fully annotated as protein-coding regions in at least two of the nine vertebrate species. We also found a subset of lincRNAs has conserved sequences in intronic regions in other species and the contribution of TEs to these alignments between lincRNAs and intronic sequences are marginal. To further explore the contribution of pseudogenized protein-coding gene to human lincRNAs, we determined which type of pseudogenization a lincRNA may originate from.

## Methods

### Genome annotation and sequence collection

cDNA sequences and genome coordinates of all the 7340 human lincRNAs annotated in the Ensembl database (release 74) were downloaded [17]. These annotated lincRNAs were identified based on chromatin features and low coding potential (i.e. for each lincRNA, no known protein domain is found, and the predicted open-reading frame, if exists, is shorter than 35% of the total length). We further removed lincRNAs that overlap with human protein-coding genes to avoid potential bias in identifying putative orthologs. A total number of 6618 lincRNAs were used in the following analysis.

Protein-coding sequences and genome annotations including non-coding gene annotations of the following nine vertebrate species were also downloaded from the Ensembl database: chimpanzee (*Pan troglodytes*; CHIM P2.1.4), orangutan (*Pongo abelii*; PPYG2), macaque (*Macaca mulatta*; MMUL_1), cow (*Bos taurus*; UM D3.1), dog (*Canis familiaris*; CanFam3.1), mouse (*Mus musculus*; GRCm38.p2), opossum (*Monodelphis domestica*; BROADO5), chicken (*Gallus gallus*; Galgal4), and zebrafish (*Danio rerio*; Zv9).

### Identification of putative orthologs of human lincRNAs in nine vertebrate species

**Fig. 1:**
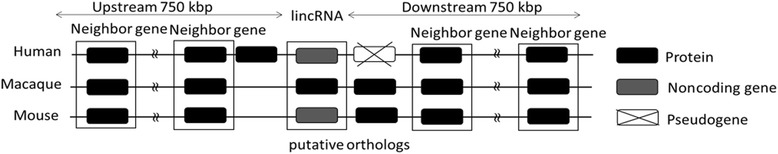
A sketch map illustrating the putative protein-coding orthologs of human lincRNAs in other species

We applied Blastn to identify matched cDNA sequences of each human lincRNA to the nine genomes. The parameters of Blastn (word size = 7, reward = 1, penalty = −1, and e-value <10^−10^) were customized to increase the sensitivity for short alignment [18]. For each Blastn match, we performed synteny analysis, which considers the order of conserved genes within the DNA regions between two species and has been shown to increase the reliability of identification of lincRNA homology region [19, 20]. The matches were constrained to contain at least one pair of conserved neighbor gene (i.e. one upstream and one downstream) within ±750 kb. We denote each of these regions as a candidate of putative ortholog (see a sketch map shown in Fig. 1 and workflow in Additional file 1: Figure S1).

To further increase the confidence of our identification, any candidate of putative ortholog identified only in one of the nine species was removed. If there were multiple potential putative orthologs, we chose the one that was present across the most number of species. In the case of a continued tie, we selected the match with the lowest e-value. If a putative ortholog overlapped with at least one protein-coding gene, we annotated the putative ortholog as a protein-coding ortholog. If a putative ortholog did not overlap with a protein-coding gene but with at least one non-coding gene (i.e. either a lncRNA or a short non-coding RNA), we annotated the putative ortholog as a non-coding ortholog. The remaining putative orthologs were annotated as intergenic orthologs. To explore whether the human long non-coding sequences are associated with the exonic regions of protein-coding genes in other vertebrates, for each protein-coding ortholog, we calculated the percentages covered by exonic, intronic, and intergenic regions of protein-coding gene. The *exon/intron/intergenic coverage* was defined as the ratio between the length of each respective region in the putative ortholog to the length of the putative ortholog.

### Investigation of TEs in putative ortholog

To determine the coverage of TEs for each identified putative orthologs, we adopted RepeatMasker 4.0.3 [21], which was downloaded from the RepeatMasker website (http://www.repeatmasker.org/). To identify TEs, we followed the criteria used by Kapusta et al. [11]: only the sequences covered by more than 10 bps of RepeatMasker-annotated TEs were regarded as those derived from TE fragments. Furthermore, to study whether the annotated exon/intron/intergenic-covered regions were originated from TEs, we calculated *TE coverage* which was defined as the percentage of sequence identified as TEs by RepeatMasker.

### Examination of potential ceRNA role of lincRNA

According to the competing endogenous RNAs (ceRNA) theory [22], an RNA transcript that has microRNA binding site can sequester microRNAs from other RNA transcripts sharing the same microRNA binding site, thus regulating their expressions. Putative ceRNA-mRNA pairs annotated in the lnCeDB database [23] were used to examine whether lincRNAs could have the potential to be ceRNAs for their putative paralog [23]. We assessed statistical significance of the observed number of ceRNA-mRNA pairs *N*_*obv*_ in the lincRNA-putative paralog pairs by using randomization by bootstrapping. Each time, we sampled *n* lincRNA-protein-coding genes from Ensembl database, where *n* is equal to the number of identified lincRNA-putative paralog pairs. The distribution of *N* was estimated given the null hypothesis that the number of ceRNA-mRNA pairs in the lincRNA-putative paralog pairs is the result of pure chance. For a one-tailed test with a rejection region in the upper tail, the bootstrap *p*-value *P(N*_*obv*_ *)* for *N*_*obv*_, was estimated by the proportion of randomized samples that contain number of ceRNA-mRNA pairs > *N*_*obv*_ . For *B* randomized datasets, we calculated the bootstrap *p*-value $P\left({N}_{obv}\right)=\frac{1}{B}\sum_{k=1}^BI\left({N}_k>{N}_{obv}\right)$, where *I(x)* is an indicator function yielding **1** if the ceRNA-mRNA pairs in *k-th* random dataset (*N*_*k*_) is more than in the original lincRNA-putative paralog pairs (*N*_*obv*_) and **0** otherwise. Here we conducted a bootstrapping analysis with *B* = 10,000.

### Bootstrapping analysis of homologous sequences

A bootstrap analysis was performed to show that the homologous sequences identified in our study are not simply artifact. By focusing on the 193 lincRNAs having protein-coding orthologs, we first shuffled each of the sequences 5000 times while controlling the di-nucleotide content. We then performed Blastn (with the same setting: word size = 7, reward = 1, penalty = −1) to align shuffled sequences to syntenic regions (with the same definition: −750 kb to +750 kb sequence with the conserved order of orthologous neighbor genes). For each shuffled sequence, we selected the alignment with the lowest Blastn *e*-value. According to our shuffling analysis, none of the shuffled sequences have a hit with blast *e*-value <10^−10^. The Blastn *e*-value distributions of shuffled sequences and original lincRNAs are shown in Additional file 2: Figure S2. The results show that our method of syntenic analysis and Blastn *e*-value is sufficient to discriminate real homologous relationships from noise.

## Results and discussion

### De novo origination and protein pseudogenization majorly contribute to lincRNA evolution

**Fig. 2:**
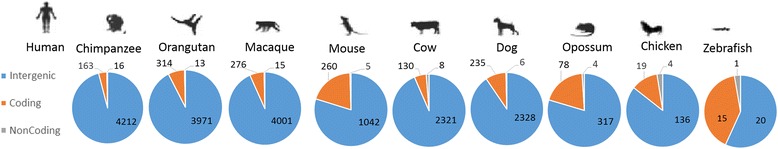
The ratios of genomic loci of putative orthologs of human lincRNAs in nine species. Most lincRNAs have putative orthologs being annotated as intergenic regions (4212 in chimpanzee, 3971 in orangutan, 4001 in macaque, 2321 in cow, 2328 in dog, 1042 in mouse, 317 in opossum, 136 in chicken, and 20 in zebrafish). Nevertheless, for a notable number of lincRNAs, the corresponding putative orthologs overlap with protein-coding genes (the numbers of coding orthologs as defined in Methods are: 163 in chimpanzee, 314 in orangutan, 276 in macaque, 130 in cow, 235 in dog, 260 in mouse, 78 in opossum, 19 in chicken, and 15 in zebrafish). The corresponding putative orthologs overlapping with non-coding genes are less comparing to intergenic regions and protein-coding gene (16 in chimpanzee, 13 in orangutan, 15 in macaque, 8 in cow, 6 in dog, 5 in mouse, 4 in opossum, 4 in chicken, and 1 in zebrafish)

We performed syntenic analysis with nine vertebrate genomes to identify putative orthologs of human lincRNAs (see Method). A putative ortholog is defined as a region that has significant sequence similarity and share the same synteny with human lincRNAs (Fig. 1). Based on the genome annotations, putative orthologs are further classified into three groups: putative intergenic orthologs, putative protein-coding orthologs, and putative non-coding orthologs (Additional file 3: Table S1, see Methods). According to the proportion of each human lincRNA group in the nine species, the majority of their putative orthologs belong to intergenic orthologs, followed by protein-coding orthologs, and only few putative orthologs are classified as non-coding orthologs (Fig. 2). Results shown in Fig. 2 reflect the relative contributions of the three lincRNA origination scenarios: de novo origination, protein pseudogenization, and duplication from lncRNA.

The predominant number of putative intergenic orthologs could be mainly contributed by de novo origination, in particular TE insertions. TEs have been found to be abundant in intergenic regions due to their jumping and amplifying ability [11, 24–26]. Together with the high TE composition of lincRNA [11, 27, 28], our observation supports that TE is one of the major factors contributing to lincRNA origination. Another possible explanation of intergenic orthologs might be incomplete annotation of non-coding RNAs. Moreover, current annotation of lincRNA could have a primate bias [29], thus leading to underestimation of the contribution of lncRNA duplication in lincRNA origination.

As the second largest group in the putative orthologs, although the ratio varies significantly across species (e.g. 4% in chimpanzee and 40% in zebrafish), the numbers of putative protein-coding orthologs are around 160 to 310 in the closer species. Among the total 6618 human lincRNAs, 297 of them (4.5%) have protein-coding orthologs in at least two vertebrates. Our results indicate some contribution of protein-coding gene pseudogenization to human lincRNAs as reported in the previous study where a lincRNA, *Xist,* retains both the syntenic context and the debris of the exon of its protein, *Lnx3* [15]. Although the number of protein-coding gene pseudogenization is less than the number of putative intergenic orthologs, the amount of putative protein-coding orthologs is more than previously documented. The reason may be previous studies focused on finding lncRNA orthologs and avoiding the high coding potential bias of the non-coding RNA sequences. Generally, the RNAs that have high coding potential, such as mRNAs and pseudogenes, are often kicked out in the lncRNA datasets in early parsing processes.

The support of protein pseudogenization is stronger when there are annotated orthologous relationships among the genes with a putative ortholog resides in different species (referred as aligned proteins). Hence, we adopted orthologous relationships in Ensembl [30] and showed that 193 of 297 (65%) of the aligned proteins are annotated as orthologous pairs. For example, human lincRNA AC004471.10 possesses aligned proteins TSSK2 in cow, ENSCAFG00000023784 in dog, ENSMMUG00000031114 in macaque, and Tssk2 in mouse, which are orthologous with each other. To sum up, the significantly greater numbers of intergenic and protein-coding orthologs than non-coding orthologs reveal the important roles of de novo origination and protein pseudogenization in lincRNA evolution.

Lastly, the number of non-coding orthologs in this study is much less than the others. One explanation is, as mentioned previously, the incomplete and biased annotation of non-coding RNAs in other species. Another reason is the relative small number of non-coding genes in the reference genome and the poor conservation of lincRNAs. The number of non-coding genes in each reference species is around 10–30% of protein-coding genes. Because protein-coding orthologs only account for 30% or less of putative orthologs, the numbers of non-coding orthologs are less than 3%. In addition, we could not identify any non-coding ortholog of lincRNA in the mouse or zebrafish genome. This result agrees with a recent study in zebrafish, where it reported merely a minority of lincRNAs show significant sequence similarity to other lncRNAs [18].

### TEs have only minor contribution in the sequence similarity between a lincRNA and its protein-coding ortholog

The conserved introns have been proposed to be a potential source of lncRNA [31]. If the corresponding orthologs of the lncRNAs locate within open reading frames (ORFs), these human lincRNAs will have higher possibility to be originated from protein pseudogenization. The investigation in the gene structure (i.e. the coverages and distributions of exons and introns) of protein-coding orthologs is needed to determine whether protein pseudogenization was involved in lincRNA evolution. To evaluate how many protein-coding orthologs could be considered as evidences of protein pseudogenization, *exon coverage*, *intron coverage,* and *intergenic coverage* were examined for each protein-coding ortholog (see Methods).

**Fig. 3:**
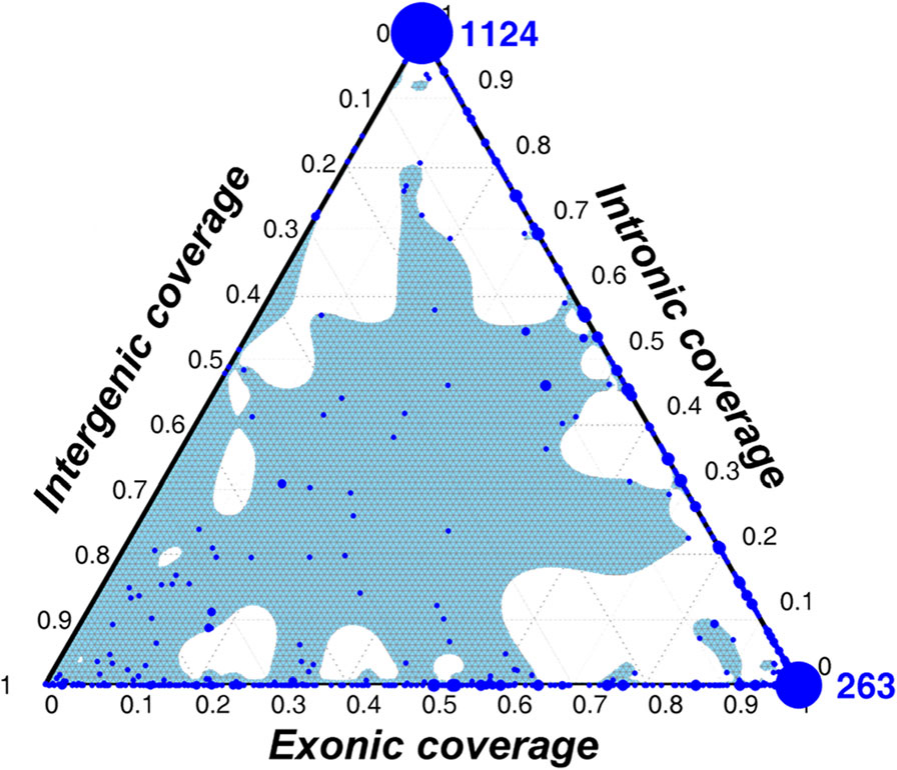
Ternary plot of *intronic coverage, intergenic coverage*, and *exonic coverage*. 1124 (52%) protein-coding orthologs that are completely intronic. Alternatively, 263 (12%) are completely exonic. The size of each dot correlates with the number of lincRNAs having this combination of *exonic coverages, intronic coverages,* and *intergenic coverages*. The blue shadow illustrates the estimated distribution

The distributions of exon coverage, intron coverage, and intergenic coverage for each protein-coding ortholog are shown in Fig. 3. Considering all the 2163 putative orthologs from the nine vertebrates, the results show 692 putative protein-coding orthologs (32%) in which *exon coverage* is greater than both *intron coverage* and *intergenic coverage*. Moreover, 263 putative protein-coding orthologs (12%) are fully located within exonic regions (i.e. *exon coverage =* 100%), which are defined as exonic orthologs in this study. On the contrary, 1124 (52%) putative protein-coding orthologs are intronic orthologs, which are completely located within intronic regions (i.e. *intron coverage =* 100%). Studies have suggested that some lncRNAs could be post-processed into small nucleolar RNAs (snoRNAs) [32], which are involved in ribosome synthesis or translation, and are usually derived from intronic sequences [33]. The hypothesis is that lncRNAs could be post-processed into snoRNAs and involved in ribosome synthesis and translation mechanism. Therefore, one of the possible explanations is that these conserved intronic orthologs might be unannotated lncRNAs.

**Fig. 4:**
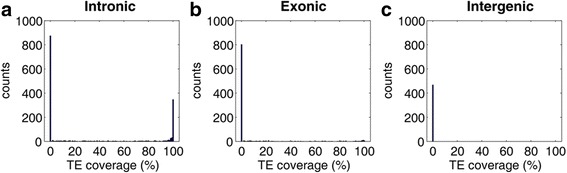
The distributions of *TE coverages* in the putative orthologs of lincRNA. **a** intronic orthologs, **b** exonic orthologs, and **c** intergenic orthologs

Furthermore, TEs could be an alternative explanation for the high intronic coverage because TEs are known to be more frequently located in introns than in exons [11, 34], and a high TE composition have been reported in lincRNAs [11, 27, 28]. Thus, we identified TE for each putative protein-coding ortholog using RepeatMasker and calculated *TE coverag*e for each putative protein-coding ortholog (see Methods). The results show that TEs covered 47% region when considering all introns of protein-coding orthologs jointly. Unexpectedly, low *TE coverages* (Fig. 4(a), average = 0.32) are observed even in the intronic orthologs. Similarly, *TE coverages* of exonic orthologs and intergenic orthologs are also low (Fig. 4(b) and (c), average = 0.07 and 0, respectively). Taking together, insertion of TEs may only contribute to a minor part of the sequence similarity between lincRNA and protein-coding orthologs.

### LincRNAs derived from duplicated pseudogenes could impact the regulation of their putative paralogs

Exonic orthologs of lincRNAs identified in our study show that a certain portion of human lincRNAs could be derived from protein pseudogenization (Fig. 3). According to the extremely low *TE coverages* in the protein-coding orthologs (Fig. 4), the results imply a minor contribution of TEs in origination of lincRNA. In particular, the *TE coverage*s are zero in the exonic orthologs of 108 lincRNAs (i.e. without TE insertion). Consequently, we asked what mechanism causes such protein pseudogenization that involved in lincRNA origination.

The GENCODE project [16] has categorized pseudogenes into three main groups based on genomic features and evolutionary mechanisms: processed pseudogenes, duplicated (also referred to as unprocessed) pseudogenes, and unitary pseudogenes. Processed pseudogenes originated from retrotransposition, which is a fraction of mRNA reversely transcribed back into the genome since they contain only exonic sequence and do not contain the upstream regulatory regions. In contrast, duplicated pseudogenes have intron-exon like genomic structures and may still maintain the upstream regulatory sequences of their parents, as a result, duplicated pseudogenes might be derived from duplication of functional genes. Lastly, since unitary pseudogene lost their coding parental gene in human and we can only find coding orthologs in other species, unitary pseudogene might have originated from accumulation of fixed disabling mutations in a coding gene [16].

**Fig. 5:**
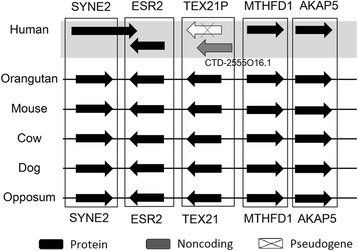
The syntenic regions across six species of lincRNA CTD-2555O16.1 which overlapped with unitary pseudogenes TEX21P. The human pseudogene TEX21P possesses homologous protein Tex21 in orangutan, mouse, cow, dog, and opossum. Protein-coding genes are indicated in black, RNA-genes in gray, and pseudogenes in white

**Fig. 6:**
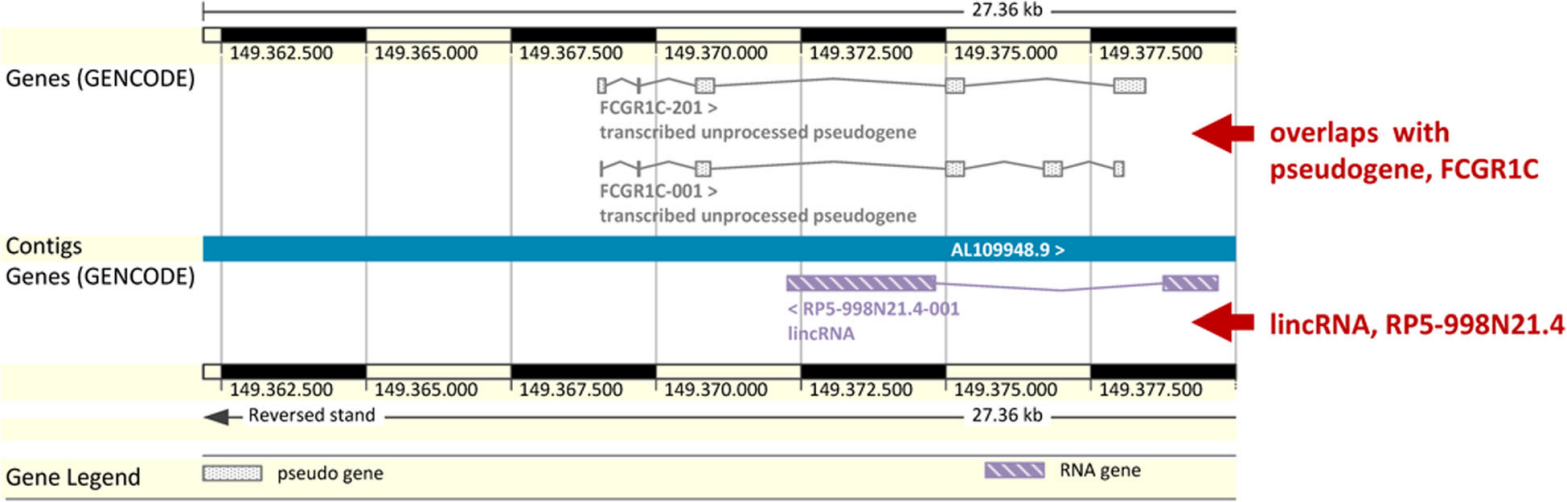
An example of lincRNAs overlapped with known pseudogenes. LincRNA RP5-998 N21.4 overlapped with the transcripts of pseudogene FCGR1C (figure modified from Ensembl genome browser Ver.74). As this particular lincRNA overlaps with the antisense of the transcribed pseudogene, the regulatory sequences involved in the lincRNA expression could be different than the ones of the processed pseudogene

Among the 108 lincRNAs having exonic orthologs, 66 of them do not have any putative paralog nor TE coverage. Moreover, 16 (24.2%) of them overlapped with pseudogenes and eight (12.1%) of them are juxtapositioned to pseudogenes, suggesting that unitary pseudogene may be their potential origination. One example is lincRNA CTD-2555O16.1, which overlapped with pseudogene TEX21P, as shown in Fig. 5. On the other hand, 42 lincRNAs have putative paralogs, that is, their aligned proteins contain at least one human ortholog (e-value less than 10^−10^). In addition, among these 42 lincRNAs, 13 (31%) overlapped with known pseudogene. Take lincRNA RP5-998 N21.4 for example (Fig. 6), its transcript overlapped with the transcript of pseudogene FCGR1C. Moreover, four lincRNAs, such as CROCCP2 and ADAM20P1, are annotated as pseudogenes of human orthologs of their aligned proteins from the Ensembl records.

A possible explanation of such relationships is that lincRNAs regulate their putative paralog by functioning as competing endogenous RNAs (ceRNAs). Based on the high sequence identity and close genomic positions between lincRNA and its putative paralog, it is possible that lincRNAs regulate their putative paralogs as ceRNAs. According to the annotation in the lnCeDB database [23], 28 lincRNA-putative paralog pairs are annotated as ceRNA-mRNA pairs. We further performed a bootstrapping analysis (see Methods) to test whether ceRNA-mRNA pairs annotated in lnCeDB database are enriched in these lincRNA-putative paralog pairs identified in this study. The significant enrichment (bootstrap *p <* 6.8 × 10^−3^) supports the proposed hypothesis that lincRNA could possibly regulate its putative paralog. However, future study is needed to find experimental support to this speculation.

Through Gene Ontology enrichment analysis, most of these putative paralogs were found to have binding functions (metal ion binding, *p* = 5.05 × 10^−9^; cation binding, *p* = 9.64 × 10^−9^; DNA binding, *p* = 1.12 × 10^−8^; nucleic acid binding, *p* = 1.88 × 10^−7^; heterocyclic compound binding, *p* = 1.80 × 10^−4^; organic cyclic compound binding, *p* = 2.65 × 10^−4^; ion binding, *p* = 6.31 × 10^−4^). In addition, these proteins are significantly associated with neuron development and eye disorder [35–37] according to the Online Mendelian Inheritance in Man (OMIM) database [38]. With conserved sequences, lincRNA could influence expression of putative paralogs by post-transcriptional regulation as endogenous siRNA or buffering effect as their decoys. Moreover, we cannot rule out the possibility that some genes are also functional in the RNA level, thus their paralogous lincRNA could also contribute to these particular functions.

## Conclusions

Protein pseudogenization is one of the scenarios of human lincRNA originations. According to the comparative genomics analyses among human and nine vertebrate species in this study, 193 of the 6614 human lincRNAs have protein-coding orthologs, which are conserved sequences in protein-coding genes of other species. Our study reveals the role of protein pseudogenization in human lincRNA origination. We anticipate that these results will bring insights to the evolutionary originations and genetic functionalities of human lincRNAs.

## Additional files

Additional file 1: Figure S1. The workflow for identifying the putative orthologs of lincRNAs in the nine species. (TIFF 256 kb)

Additional file 2: Figure S2. The Blastn *e*-value distributions of shuffled sequences and 193 lincRNAs (see Methods). (PDF 5 kb)

Additional file 3: Table S1. The putative orthologs of human lincRNAs in the nine analyzed species. (XLSX 1397 kb)

## Abbreviations

ceRNA: competing endogenous RNA
lincRNA: long intergenic noncoding RNA
lncRNA: long non-coding RNA
ORF: Open reading frame
TE: Transposable element
