# Supplementary figures and images for: Comparative genomic analyses highlight the contribution of pseudogenized protein-coding genes to human lincRNAs
Source: BMC Genomics. 2017 Oct 16;18:786. doi: 10.1186/s12864-017-4156-x (PMC5644071; doi:10.1186/s12864-017-4156-x)

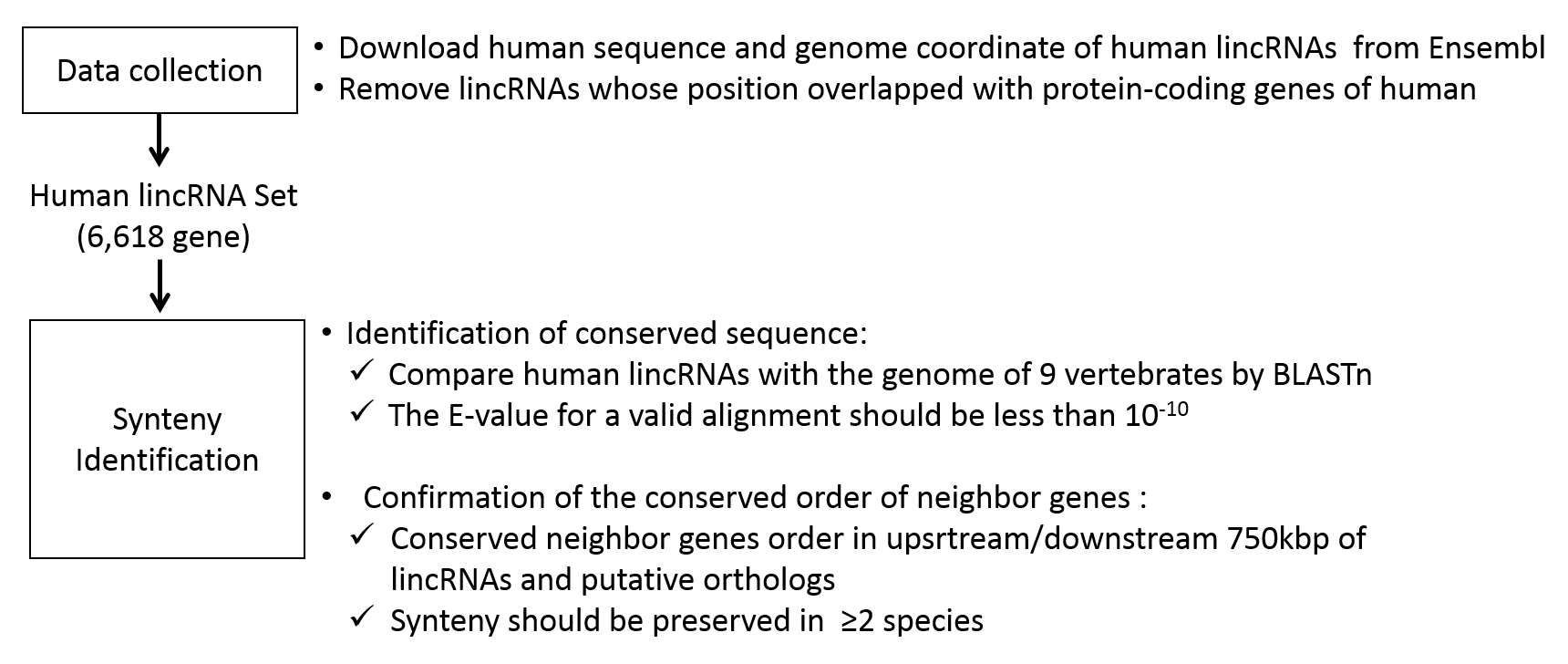

Supplement: Supplementary file 1 — The workflow for identifying the putative orthologs of lincRNAs in the nine species. (TIFF 256 kb) [file 12864_2017_4156_MOESM1_ESM.tif]

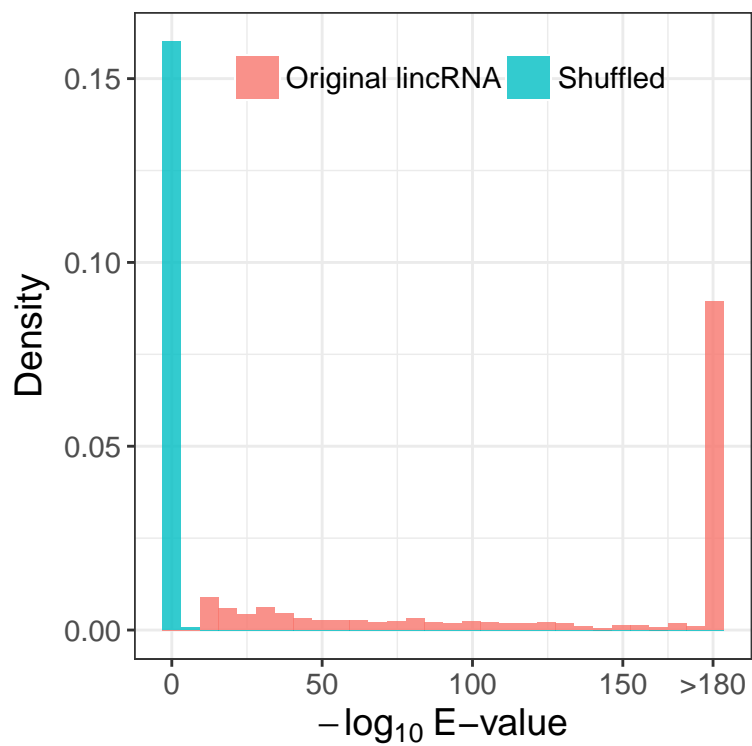

Supplement: Supplementary file 2 — The Blastn e-value distributions of shuffled sequences and 193 lincRNAs (see Methods). (PDF 5 kb) [file 12864_2017_4156_MOESM2_ESM.pdf]
